# Strain specific behavioral and physiological responses to constant light in male CBA/J and CBA/CaJ mice

**DOI:** 10.5935/1984-0063.20200096

**Published:** 2021

**Authors:** Hannah V Deane, Holly A Concepcion, Avery E Gatewood, Janessa Quintana, Joseph A Seggio

**Affiliations:** Bridgewater State University, Department of Biological Sciences - Bridgewater - MA - United States.

**Keywords:** Glucose Tolerance Test, Circadian Rhythm, Locomotion, Light, Mice, Inbred Strains

## Abstract

**Objective:**

Being visually impaired increases the likelihood of sleep disorders and altered behavior. This study investigated physiological and behavioral differences in two similar mice substrains when exposed to constant light (LL) - CBA/J with retinal degeneration and CBA/CaJ mice (no retinal degeneration).

**Material and Methods:**

Male CBA/J and CBA/CaJ mice were placed into a 12:12 light:dark cycle or constant light (LL). Open field behavior, metabolic markers, and home-cage circadian activity were observed.

**Results:**

CBA/CaJ mice have greater circadian period lengthening, increased weight gain, reduced glucose, and increased novelty-induced locomotor activity in LL, compared to CBA/J mice. LL reduced thyroid hormone and insulin in both substrains.

**Discussion:**

While several baseline substrain differences were elucidated, CBA/CaJ mice were more effected by the exposure to LL than the blind CBA/J mice. These results illustrate that LL causes alterations in physiology and behavior and that circadian photoreceptivity might contribute to these effects.

## INTRODUCTION

Previous studies have shown that exposure to constant light (LL), light-at-night, through increased screen time (e.g., TVs, tablets, and smartphones), or night-shift work, can disrupt the circadian rhythm, leading to altered metabolism, hormonal rhythms, and increased anxiety-like and depression-like behaviors^1-4^. The metabolic and behavioral abnormalities caused by continuous light exposure can be attributed to altering the amount of light exposure^5^ and the inability to synchronize to environmental and photic cues^6^.

Previous work has shown that individuals can have altered biological clock function if they are visually blind or experience retinal degeneration compared to sighted individuals in both animal and human studies^7,8^. One commonly used mouse model in biomedical and behavioral studies, the CBA/J mouse, carries the *Pde6brd1* mutation, which causes retinal degeneration by wean age, while other CBA substrains (CBA/N and CBA/CaJ) do not. This mutation causes degeneration of rhodopsin photoreceptor cells (used for vision), but does not affect melanopsin photoreceptor cells within the retina, which are used for circadian entrainment to a light:dark cycle (LD) cycle, allowing mice with retinal degeneration the ability to synchronize to a LD cycle. While rhodopsin is not required to synchronize to the LD cycle, melanopsin deficient mice with intact visual photoreceptors are able to entrain and only show mild deficits in circadian photosensitivity, indicating that there may be some overlap between these two photic pathways^9^.

For the CBA substrains, CBA/CaJ and CBA/N mice have normal circadian and visual photosensitivity, while CBA/J mice exhibit reduced circadian photosensitivity starting at approximately 10-weeks of age, quantified by reduced phase shifts to light pulses^10-12^.

This study aims to examine metabolic and physiological substrain-specific differences between CBA/CaJ (without retinal degeneration) and CBA/J (with retinal degeneration) mice when exposed to constant room-level lighting (LL). The CBA/CaJ substrain is not visually blind, while the CBA/J substrain experiences retinal degeneration.

## MATERIAL AND METHODS

### Animals

All animal studies were carried out with approval from Bridgewater State University’s Institutional Animal Care and Use Committee (IACUC). Thirty-four male CBA/J and CBA/CaJ (Jackson Laboratories, Bar Harbor, ME, USA) mice were purchased at approximately 9 weeks of age. Upon arrival, were housed individually and placed in a 12:12 h LD cycle (lights on 0600-1800H; lights off 1800-0600h, ceiling LED lights ~150 lux) with regular chow (LabDiet 5001, St. Louis, MO, USA) and water *ad libitum*. Circadian rhythms were measured using infrared home-cage sensors (StarrLife Sciences, Oakmount, PA, USA) as previously described^13^. After a one-week acclimation period, half of each substrain of mice were placed into constant light (LL) cycle while the other half remained in a LD cycle at room level lighting. Four total groups have been set up in a 2 x 2 design: (1) CBA/J + LL (J/LL) (n=8); (2) CBA/J + LD (J/LD) (n=9); (3) CBA/CaJ + LL (CaJ/LL) (n=9); and (4) CBA/CaJ + LD (CaJ/LD) (n=8). Weekly measurements of food consumption and body mass were recorded. All the behavioral and physiological assays were performed at Zeitgeber Time (LD animals) or Circadian Time 6 (LL animals), which is the middle of the inactive period for both sets of animals so that all mice were tested in the light and at the same relative activity phase to each other.

### Open field

After 6 weeks of exposure to LD or LL, an open field test was performed using the SmartCage™ system (AfaSci Inc., Redwood City, CA, USA). The open field assay was performed to assess anxiety-like and explorative behaviors, as previously described^14^.

### Physiological tests

Two weeks following the open field assay, a glucose tolerance test (GTT) was performed to determine the glucose sensitivity of each individual. After a 12-hour fast, a small prick was made at the tip of the tail and a baseline blood glucose was measured by One-Touch Ultra-2 glucose monitors. An intraperitoneal injection of 2g/kg of glucose was administered to each mouse and blood glucose levels were measured post-injection at 30, 60, and 120 minutes post-injection.

After one week of recovery from the GTT, mice were fasted for 4 hours, and then euthanized via CO^2^ narcosis. Whole blood was collected, allowed to clot, and centrifuged at 2,000g at 4˚C for 20 minutes to obtain serum. Blood serum was stored at-80˚C. After storage, serum used to measure insulin (Ultra-Sensitive Mouse Insulin ELISA Kit, Crystal Chem Inc., Downers Grove, IL, USA), thyroid stimulating hormone (TSH) (MBS777023, Mouse Thyroid Stimulating Hormone, TSH ELISA Kit, MyBioSource, San Diego, CA, USA) and free thyroxine (fT4) (MBS765283, Mouse Free Thyroxine ELISA Kit, MyBioSource).

Simultaneous with blood collection, frontal lobe sections (1mm^3^) were manually dissected and stored immediately in-80˚C. After storage, tissue homogenates were created as previously described^2^, and the supernatant was tested for testosterone (MBS288265, General Testosterone ELISA Kit, MyBioSource).

### Statistical analysis

Circadian period and locomotor activity were calculated using Clocklab’s (Actimetrics, Wilmette, IL, USA) automated chi-square and bout analysis functions. Area under the curve (AUC) was calculated for each mouse to assess glucose clearance overtime for the GTT. Two-way ANOVAs with Tukey *post-hoc* pairwise comparisons were used to assess the light cycle and substrain differences in each group for all metabolic and behavioral assays.

## RESULTS

### Circadian locomotor activity

**Figure 1:**
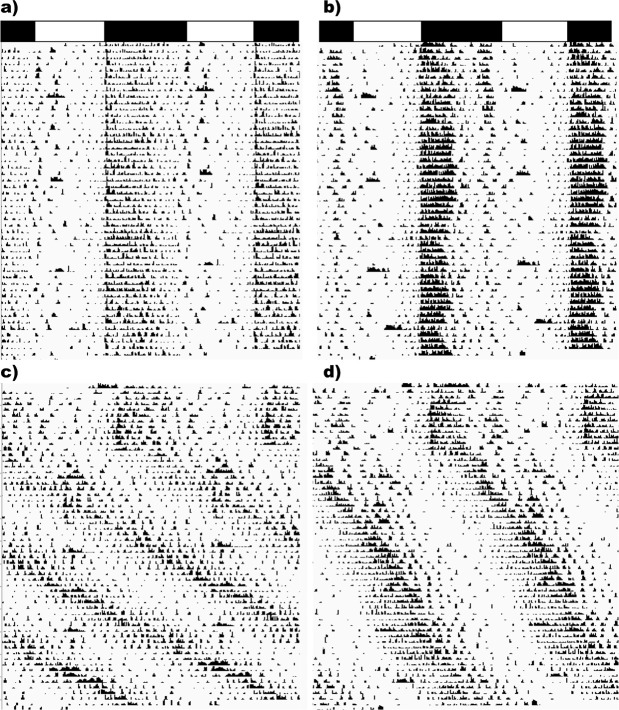
Representative Actograms. a) CBA/CaJ + LD, b) CBA/J + LD, c) CBA/CaJ + LL, d) CBA/J + LL.

The means and SEM of all of the circadian locomotor activity parameters analyzed are summarized in Table 1 and representative actograms are provided in Figure 1. A substrain by light cycle interaction was found for the circadian period. While all animals from both genotypes entrained to the standard 12:12LD cycle, CBA/CaJ mice in LL exhibited longer periods than CBA/J mice (F_1,30_=247.51, *p*<0.001). LL produced reduced rhythm power (F_1,30_=30.53, *p*<0.001) and alpha (F_1,30_=8.48, *p*=0.009) compared to LD, but there were no substrain differences present. For daily home-cage activity, LL produced reductions in overall activity (F_1,30_=9.52, *p*=0.006), counts per activity bout (F_1,30_=5.96, *p*=0.025), and peak activity (F_1,30_=13.61, *p*=0.002), but not length of activity bout (F_1,30_=0.39, *p*=0.54). Regardless of lighting cycle, CBA/CaJ mice exhibited an increased number of locomotor bouts per day compared to CBA/J (F_1,30_=8.10, *p*=0.010).

**Table 1:** Circadian actograms reveal increased period length, and reduced power, activity counts per day, and circadian peak in animals exposed to LL independent of strain. CBA/CaJ mice in LL experience greater period lengthening compared to CBA/J in LL. Values with letters (a,b,c) indicates significant pairwise comparison at p<0.050.

Circadian locomotor activity								
Genotype	Cycle	Period	Power	Activity	Length	Counts	Peak	Bout/day
CaJ	LD	24 ± 0.00^a^	3016.96 ± 464.11^a^	25.15 ± 4.92^a^	40.96 ± 8.05	314.05 ± 101.57^a^	6.71 ± 0.57^a^	11.47 ± 1.00^a^
CaJ	LL	25.64 ± 0.06^b^	874.68 ± 38.74^b^	15.82 ± 1.49^b^	32.94 ± 1.54	152.64 ± 16.51^b^	5.19 ± 0.33^b^	13.75 ± 0.69^a^
J	LD	24 ± 0.00^a^	2767.91 ± 432.25^a^	28.26 ± 4.14^a^	45.83 ± 5.14	347.42 ± 64.97^a^	7.53 ± 0.61^a^	10.75 ± 0.75^b^
J	LL	24.61 ± 0.04^c^	1237.57 ± 253.72^b^	18.13 ± 2.42^b^	43.80 ± 3.95	226.08 ± 35.85^b^	5.75 ± 0.44^b^	10.56 ± 0.45^b^

### Open field

The means and SEM of all of the open field parameters analyzed are summarized in Table 2. An interaction was uncovered differences in active time (F_1,29_=4.26, *p*=0.049). Whereas, CBA/CaJ mice in LL exhibited increased active time in the open field (*p*=0.010), no differences were found between LD and LL in CBA/J mice (*p*=0.95). Baseline substrain differences were found for distance traveled (F_1,30_=11.07, *p*=0.018, CBA/CaJ > CBA/J), velocity (F_1,30_=8.85, *p*=0.006, CBA/CaJ > CBA/J), and time spent in the center zone (F_1,30_=11.63, *p*=0.002, CBA/CaJ<CBA/J), but LL had no effects on these variables. No differences were present for rearing behavior (F_1,30_=0.16, *p*=0.70).

**Table 2:** CBA/CaJ mice increased active time in LL but no differences were found for CBA/J mice. Regardless of light cycle, CBA/CaJ mice exhibited increased distance traveled and velocity, as well as reduced center time zone compared to CBA/J mice. Values with letters (a,b,c) indicated significant pairwise comparison at p<0.050.

Open field							
Genotype	Cycle	Center zone time (min)	Active time (min)	Distance (cm)	Velocity (cm/s)	Rears	Total rotations
CaJ	LD	0.94 ± 0.15^a^	7.55 ± 0.66^a^	1230.53 ± 94.24^a^	3.17 ± 0.16^a^	63.50 ± 13.83	10.69 ± 0.96^a^
CaJ	LL	0.97 ± 0.11^a^	9.09 ± 0.20^b^	1438.56 ± 61.85^a^	3.27 ± 0.12^a^	74.22 ± 16.34	15.38 ± 1.61^b^
J	LD	1.32 ± 0.09^b^	8.20 ± 0.13^c^	1123.81 ± 53.68^b^	2.83 ± 0.15^b^	69.89 ± 12.47	13.80 ± 1.44^a^
J	LL	1.34 ± 0.14^b^	8.43 ± 0.11^c^	1202.18 ± 59.41^b^	2.86 ± 0.09^b^	90.50 ± 8.53	15.84 ± 1.15^b^

### Physiological responses

**Figure 2:**
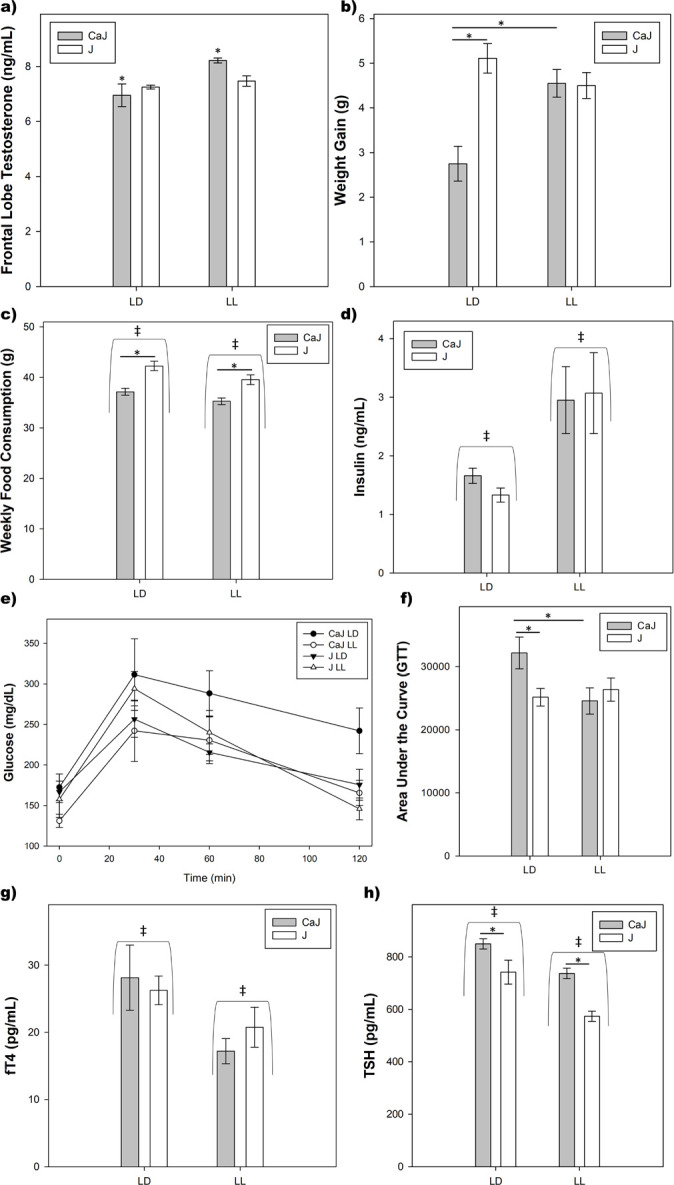
Physiological Characteristics. a) Frontal Lobe Testosterone. CBA/CaJ mice in LL exhibited increased testosterone compared to LD controls. CBA/J mice had no such increase in LL. b) Weight Gain. In LD, CBA/CaJ mice exhibited lower weight gain compared to CBA/J mice, but were not different in LL. LL exposure lead to increased weight gain in CBA/CaJ mice, but not in CBA/J mice. c) Weekly Food Consumption. Both substrain (CBA/CaJ < CBA/J) and light cycle (LD > LL) differences were found. d) Serum Insulin. LL produced increases to insulin regardless of substrain. e) Glucose levels over time for the Glucose Tolerance Test (GTT). f) Area Under the Curve for GTT. CBA/CaJ mice in LD exhibited increased glucose levels over time compared to CBA/J mice in LD and CBA/CaJ mice in LL. g) Serum Free Thyroxine (fT4). Reduced fT4 was observed in LL regardless of substrain. h) Serum Thyroid Stimulating Hormone (TSH). LL led to reductions in TSH in both substrains. CBA/CaJ mice exhibited increased TSH compared to CBA/J.

An interaction was uncovered differences for frontal lobe testosterone (F_1,30_=6.25, *p*=0.019). While no differences were found between LD and LL for CBA/J mice (*p*=0.075), CBA/CaJ in LD exhibited lower frontal lobe testosterone compared to CBA/CaJ in LL (*p*=0.001) (Figure 2A).

A genotype by lighting condition interaction was uncovered for weight gain (F_1,30_=6.93, *p*=0.013). In LD, CBA/CaJ mice are smaller than CBA/J mice (*p*=0.006). LL led to an increase in body mass for CBA/CaJ mice (*p*=0.011), but not CBA/J mice (*p*=0.99) (Figure 2B). There were substrain differences (F_1,30_=5.33, *p*<0.001, CBA/CaJ<CBA/J) and light cycle differences (F_1,30_=9.13, *p*=0.005, LD>LL) for food consumption, but no interaction was present (Figure 2C). LL produced increases to serum insulin levels (F_1,30_=11.89, *p*=0.002), but there were no substrain differences or interaction present (Figure 2D). Baseline glucose was higher in LD compared to LL (F_1,30_=10.42, *p*=0.003). An interaction was found for glucose tolerance area under the curve (F_1,30_=4.82, *p*=0.036), whereas CaJ mice in LD exhibited increased area under the curve compared to CaJ in LL (*p=*0.050), but no differences were found for CBA/J mice (*p=*0.97) (Figures 2E and 2F). LL led to decreases in fT4 (F_1,30_=6.89, *p*=0.014) regardless of substrain (Figure 2G). Both substrain (F_1,26_=16.28, *p*<0.001, CBA/CaJ>CBA/J) and light cycle (F_1,26_=19.10, *p*<0.001, LD>LL) differences for TSH levels were also found (Figure 2H).

## DISCUSSION

In this study, we report circadian, behavioral, and physiological substrain differences between CBA/CaJ and CBA/CJ mice in response to LL exposure. First, CBA/CaJ mice experience a longer period lengthening in LL compared to CBA/J mice, which is independent of other circadian activity parameters. There may be connections among light intensity, circadian photoreception, and the length of period changes in response to LL. Melanopsin knock-out mice show blunted period lengthening in response to LL compared to wild-type controls^15^. These results may be partly due to the finding that individuals experiencing blindness may receive less light to the SCN and the hypothalamus^16^. Other studies have also shown that a greater degree of light intensity is correlated with increased period lengthening in sighted animals^17^. Previous work has also shown that CBA/J mice exhibit reduced circadian photosensitivity compared to sighted CBA mouse strains^10,11,12^. Despite having reduced circadian photosensitivity, CBA/J mice exhibit increased VIP and light-induced c-fos induction within the SCN^18^ and intact melanopsin^19^. These incongruent results may be due to increased GABA signaling which accompanies VIP within the SCN core^18^ or to outer retina degeneration^19^, although neither hypothesis has been tested directly. Additionally, LL reduces the amount of VIP within the core of the SCN^20,21^, although it is unknown whether this phenomenon occurs in CBA/J mice.

The reduced period lengthening found in CBA/J mice may be due in part to their decreased circadian photosensitivity and their outer retina degeneration. Interestingly, this reduction in circadian photosensitivity may be substrain specific to CBA mice as C57BL/6 mouse substrains exhibit similar circadian responses to light whether sighted or mutated for retinal degeneration^22^. The genetic mutation leading to retinal degeneration in C57BL/6 mice is different from the one found in CBA/J mice, which may also explain the difference in circadian light responsiveness seen between these two mouse models.

CBA/CaJ mice exposed to LL exhibited increased activity time in the open field compared to controls under the standard LD cycle. Other studies have shown that the novelty-induced locomotor activity responses to LL are strain and species specific^1,2^. LL also seems to increase locomotor activity parameters of other behavioral assays, such as the number of transitions between zones in the light-dark box and the elevated-plus maze, sometimes without affecting the main indicator of anxiety-like behaviors (i.e., time spent in the dark zone or closed arms) in rhythmic animals^1,2^.

Studies where animal models become arrhythmic due to LL also report impairments to mood regulation^23,24^, similar to impairments found in arrhythmic animals due to other forms of circadian disruptions^25^. These results raise the question of whether the effects of LL on emotionality behaviors are due to the circadian desynchrony itself, alterations to the amount of light exposure the animals are experiencing or a combination of both. LL is an interesting experimental circadian paradigm, as animals can be arrhythmic, exhibit splitting, or maintain behavioral rhythmicity when exposed to LL; animals that are arrhythmic exhibit robust *per1:GFP* SCN neuronal rhythms that are out of phase with each other, splitting animals have SCN neurons oscillating in antiphase with each other, while rhythmic animals exhibit robust synchronous SCN neuronal rhythms that correlate with the onset of the behavioral rhythm^26^. As such, it is difficult to parse the reasons why similar responses to behavior occur in both rhythmic and arrhythmic animals in LL. Interestingly, reducing the amount of light exposure can lead to deficits in mood regulation, similar to what is found in animals under LL.

Previous work using T=20 cycles where there are variations to the amount of light exposure (but no entrainment or arrhythmia in the animals) leads to alterations in mood regulation similar to what is found in LL^27^. Additionally, a recent study from our lab has also shown behavioral differences (novelty-induced activity and anxiety-like behaviors) in response to a T=21 cycle can occur in mice where behavioral entrainment was possible to the light cycle^28^. This result implies that altering the amount of light exposure (whether increased or decreased) to the circadian timing system and to other brain regions can modulate emotionality, whether the animals are rhythmic or arrhythmic.

In this study, the lack of effects of LL on open field behaviors in CBA/J mice and species may be partially due to reductions in circadian photosensitivity compared to CBA/CaJ mice and other strains. Nevertheless, recent work has also shown that photic signaling via the intrinsically photosensitive retinal ganglion cells-SCN pathway is not involved in light-mediated mood regulation; rather this regulation is controlled by the perihabenular nucleus (PHb) within the thalamus, which itself exhibits rhythmicity^5^. It was further demonstrated that a T=7 cycle abolishes the rhythmicity of PHb, which leads to the altered behavior seen in animals with intact melanopsin, whereas animals with no melanopsin do not show deficits to behavior^5^. These results imply that abolishing rhythmicity to areas controlling certain behaviors, regardless of lighting cycle (whether they have direct SCN inputs or not) may mediate those behavioral responses to circadian alterations. Future studies investigating the link among emotionality, rhythmicity, and responsiveness to light, continuous or otherwise, would be of great interest.

Additionally, testosterone was increased in CBA/CaJ mice, but not CBA/J mice, in LL. Testosterone is linked to increased levels of ambulation and anxiety-like behaviors when animals are subjected to behavioral tests including the open field and is also linked to emotional behaviors^29^. Reductions in testosterone through gonadectomy can reduce open field movement^30^, while exogenous administration can lead to increased activity within the open field^31^. Exposure to light has also been shown to prevent the reductions in testosterone caused by sleep deprivation in humans^32^. The increase in active time in CBA/CaJ mice may be in part due to the increased testosterone levels found in those mice exposed to LL.

CBA/CaJ mice experienced increased weight gain and reduced glucose levels in LL compared to CBA/J mice. Previous rodent studies reported that weight gain in response to LL can be substrain and species-specific as some studies report weight gain^2^ and others observe none^3,33^ in animals with intact visual receptors. Surprisingly, CBA/CaJ mice exhibited reduced glucose levels when exposed to LL with corresponding increases to serum insulin; other studies usually report that circadian disruption leads to both hyperinsulinemia and hyperglycemia, which are symptoms of a type 2 diabetic-like state (as summarized by Vinogradova et al. (2009)^33^). This result may be the manifestation of baseline hyperglycemia and hyperinsulinemia within CBA/CaJ mice as found here (in LD elevated glucose and insulin compared to CBA/J) and elsewhere^34^. Another possibility is that the oscillations of glucose and insulin may be at different phases in LL compared to the behavioral rhythm, which may explain the lower glucose levels, as was seen in an example of a type 2 diabetic rodent model experiencing simulated jet-lag^13^. Previous work also illustrates that type 2 diabetes itself can affect the rhythmicity of insulin^35^ and melatonin^36^, potentially leading to peripheral oscillators being at a different phase compared to behavioral rhythms.

Meanwhile, both CBA substrains in this study had reduced TSH and reduced fT4 in response to LL. Previous work in non-CBA substrains has consistently reported that LL leads to reductions in TSH and increases to fT4, which indicates hyperthyroid symptoms^2,3,33^. Conversely, reductions in both TSH and fT4, as seen in the CBA substrains, are indicative of secondary hypothyroidism. This current result (decreases in both fT4 and TSH) may be due to the CBA substrains exhibiting a mild form of thyroiditis. Previous work has reported that CBA mice are more susceptible to thyroiditis than other substrains^37^, although we did not investigate the size or structure of the thyroid gland in this study. These results also indicate that the effects of light exposure on thyroid-related hormone levels may be mediated through the circadian timing system and not through the visual pathway, as both the sighted and blind substrain experienced the same reductions in hormone secretion.

It is worth noting that the results presented in these experiments are from a single time-point only, rather than over the course of the circadian cycle. As such, this raises the question of whether or not the animals are in phase with their behavioral cycles and their physiological rhythms. If the behavioral and physiological assays were conducted at a different time-point, different results may have been obtained. LL may lead to desynchrony even if animals exhibit a stable free-running rhythm, while physiological pathologies can manifest in altered rhythms even if animals are entrained, as previously mentioned. For example, LD and LL animals exhibit numerous differences in insulin and glucose responses during the subjective day, but fewer differences during the subjective night^38^. Additionally, rhythmic LL animals tested along the daily cycle show blunting or arrhythmicity of some hormonal rhythms compared to animals in DD or LD^39,40^. Nevertheless, this study adds to the body of evidence that illustrates that exposure to LL can lead to altered behavioral and metabolic outcomes particularly during the subjective day, similar to what was found in previous work.

In summary, there are several substrain-specific responses observed in the CBA/CaJ and CBA/J mice in response to LL. We uncovered several instances where LL altered behavior or physiology in the sighted CBA/CaJ mice only. Either both substrains were equally affected by LL, as in the case of thyroid-related hormones and insulin levels, or it was the CBA/CaJ substrain, which was more affected by LL than CBA/J mice (weight gain, glucose levels, open field activity, and testosterone). Overall, CBA/CaJ mice are more susceptible to the effects of LL perhaps due in part to their ability to visually perceive light compared to CBA/J mice, and their reduced circadian photosensitivity. Additional studies that investigate differences in circadian rhythm and sleep function between blind and sighted individuals will be of enormous import as they can provide a foundation for future clinical and basic scientific studies.
